# Functional Analysis of Two Odorant-Binding Proteins, MaltOBP9 and MaltOBP10, in *Monochamus alternatus* Hope

**DOI:** 10.3389/fphys.2020.00317

**Published:** 2020-04-15

**Authors:** Dong-Zhen Li, Xiao-Feng Huang, Rui-Nan Yang, Jing-Yuan Chen, Man-Qun Wang

**Affiliations:** ^1^Hubei Insect Resources Utilization and Sustainable Pest Management Key Laboratory, College of Plant Science and Technology, Huazhong Agricultural University, Wuhan, China; ^2^Hubei Academy of Forestry, Wuhan, China

**Keywords:** cellular localization, fluorescence competitive binding assay, *Monochamus alternatus*, odorant-binding protein, insect olfaction

## Abstract

Odorant-binding proteins (OBPs) are important for the perception of chemical signals by insects. Effective pest management strategies can be developed by understanding the host location mechanism and the physiological functions of OBPs in olfactory detection. In this study, we cloned two OBPs from *Monochamus alternatus*, where MaltOBP9 was highly expressed in multiple insect tissues and MaltOBP10 was highly expressed in the female antenna according to the results of qRT-PCR. The recombinant proteins were successfully purified *in vitro*. Immunocytochemistry indicated the high expression of MaltOBP9 and MaltOBP10 in the sensillum lymph of sensilla basiconica, sensilla trichodea, sensilla auricillica, and sensilla chaetica, thereby demonstrating their broad participation in semiochemical detection. Both proteins were localized in the inner cavity of mechanoreceptors and they exhibited broad binding abilities with volatiles from pine bark according to fluorescence competitive binding assays. Due to its broad binding ability and distribution, MaltOBP9 may be involved in various physiological processes as well as olfactory detection. MaltOBP10 appears to play a role in the fundamental olfactory recognition process of female adults according to its broad binding ability. These findings suggest that OBPs may have various physiological functions in insects, thereby providing novel insights into the olfactory receptive mechanism.

## Introduction

The special and sensitive olfactory system of insects perceives infochemicals in the environment, such as plant volatiles and pheromones, and it affects the lifecycle of insects by regulating behaviors, such as foraging, aggregation, mating, spawning, dispersion, and defense (Zwiebel and Takken, 2004; Leal, 2013; Brito et al., 2016). Olfactory sensation is an extremely complicated pathway that involves diverse proteins, including odorant-binding proteins (OBPs), chemosensory proteins (CSPs), odorant degrading enzymes (ODEs), odorant receptors (ORs), and sensory neuron membrane proteins (SNMPs) (Meiners et al., 2003; Benton et al., 2007; Jin et al., 2008; Venthur et al., 2015; Zheng et al., 2016). OBPs are considered to operate in the first step of chemical cue reception by combining and transporting lipophilic odorants across the sensillum lymph to ORs in the dendritic membranes of olfactory receptor neurons (Krieger et al., 1996; Laughlin et al., 2008; Brito et al., 2016). The signature of the classic OBPs is a pattern comprising of six cysteines in conserved positions, which are connected in the native protein by three interlocking disulfide bridges (Lescop et al., 2009; Zhuang et al., 2014; Brito et al., 2016). An internal hydrophobic cavity is used for binding odorants (Mao et al., 2010; Spinelli et al., 2012). Other non-classical OBPs have also been identified, including minus-C OBPs, which contain only four conserved cysteines, and plus-C OBPs, which contain more than six conserved cysteines (Zhou et al., 2004; Forêt and Maleszka, 2006; He et al., 2016).

Numerous OBP genes have been identified in different insects and it is recognized that many OBPs are specifically expressed in the antennae, whereas some OBPs are also expressed in other tissues, thereby indicating that OBPs might be involved with different functions (Li et al., 2005; Pelosi et al., 2006; Pitts et al., 2011; Jeong et al., 2013; Mastrobuoni et al., 2013; De Biasio et al., 2015; Ma et al., 2016). In *Batocera horsfieldi* Hope, BhorOBP1, BhorOBP2, and BhorOBP3 are expressed in the antennae, but also in the wings, legs, abdomens, maxillary palps, and labial palps (Li et al., 2012). These differences in distribution may be related to their specific features (He et al., 2011; Ma et al., 2016; Wang et al., 2019). Previous studies have shown that OBPs are expressed selectively in different types of sensilla on the antenna, which are considered the minimum functional units for chemoreception. In *Adelphocoris lineolatus*, AlinOBP1 is located on long trichoid sensilla and medium length sensilla basiconica, whereas AlinOBP13 is located on short basiconica sensilla (Gu et al., 2011b; Sun et al., 2014). In general, basiconic sensilla are considered to be sensitive to plant odors, whereas long trichoid sensilla respond to sex pheromones (Lopes et al., 2002). These specific differences in expression suggest possible functional connections or differences.

Fluorescence competitive binding assays are effective for studying the binding characteristics and functions of OBPs in many insects *in vitro* (Maïbèche-Coisne et al., 1997; Zhang et al., 2011; Li et al., 2015; Zheng et al., 2016). In general, OBPs show higher binding affinities with ligands in neutral conditions than acid conditions *in vitro* (Wogulis et al., 2006; Mao et al., 2010; Leite et al., 2011; Li et al., 2015). In addition, several protein structure studies demonstrated that the pH can influence the conformation of OBPs, and thus it was suggested that OBPs bind odors at a neutral pH and release them in the acid environments around dendritic membranes in neurons (Sandler et al., 2000; Wogulis et al., 2006; Damberger et al., 2007; Zhou et al., 2009; Leite et al., 2011). Moreover, the classical fluorescence competitive binding assay is the key technique for insect reverse chemical ecology by targeting OBPs to screen behaviorally active compounds of insects (Tsitsanou et al., 2012; Jayanthi et al., 2014; Brito et al., 2016). Using insect reverse chemical ecology methods, it was shown that (+)-β-pinene binds with DhelOBP21 and it is attractive to *Dastarcus helophoroides* (Yang et al., 2017), which is a major biological control agent against *Monochamus alternatus* Hope (Wei et al., 2009).

The Japanese sawyer beetle *M. alternatus* Hope (Coleoptera: Cerambycidae) is a serious pest and a major vector of the pine wood nematode *Bursaphelenchus xylophilus* (Steiner et Buhrer) Nickle (Nematoda: Aphelenchoididae). *B. xylophilus* causes a devastating pine disease (Kobayashi et al., 1984). A previous study showed that among the volatiles in pine bark, monoterpenes substances such as α-pinene, β-pinene, camphene, and myrcene play roles as directional lures of *M. alternatus* (Fan et al., 2007). Studying the physiological functions of OBPs is beneficial for understanding the olfactory recognition process in *M. alternatus* and screening behaviorally active compounds by insect reverse chemical ecology by targeting OBPs. In our previous work, antennal transcriptome of *M. alternatus* has been assembled and analyzed, and 25 ORFs of OBPs genes have been identified, and the molecular characterization and volatile binding properties of a few of these OBPs have been conducted (Wang et al., 2014). Gao investigated the binding affinities of MaltOBP3 and MaltOBP5 which is classic OBP and minus-C OBP respectively, and found that these two OBPs share relatively high-affinity with some compounds such as (-)-limomene, α-terpinolene and camphor (Gao and Wang, 2015). Another research showed that MaltOBP13 exhibited a high binding affinity to most pine volatiles (Li et al., 2017). However, to make a deeper understanding of the roles that OBPs play in the life of *M. alternatus*, researches about the expression and ligand-binding properties of other OBPs are required. In this study, detail analysis of two OBPs, MaltOBP9 and MaltOBP10, were performed. MaltOBP9 (NCBI accession number KF977562), which is expressed in a broad range of *M. alternatus* tissues, and MaltOBP10 (NCBI accession number KF977563), which is specifically expressed in the antennae, were cloned, expressed and purified the proteins *in vitro* to research their potential olfactory functions in this study. The specific localizations of MaltOBP9 and MaltOBP10 in the antennal sensilla of *M. alternatus* were studied by immunoelectron microscopy to investigate their olfactory functions. Fluorescence binding assays were conducted to study the binding characteristics of MaltOBP9 and MaltOBP10 with 17 volatiles. The results showed that MaltOBP9 and MaltOBP10 had broad ligand-binding capacities with these volatiles, including α-pinene, β-pinene, camphene, and myrcene. Thus, MaltOBP9 and MaltOBP10 appear to play roles in the fundamental olfactory recognition process as well as being involved in other physiological processes.

## Materials and Methods

### Insects

Dead *Pinus massoniana* trees that had natural infestations with *M. alternatus* larvae were harvested in Yichang, Hubei, P. R. China (110°29′E, 30°70′N) in November and December, 2012. No specific permits were required for the field studies. The sampling locations were not privately owned or protected in any way, and this field study did not involve endangered or protected species. Trees were placed in indoor cages in April 2013 and emerging *M. alternatus* adults were collected daily until early August. Adults were reared on *P. massoniana* twigs in a cage at 25°C, with a photoperiod comprising illumination for 14 h and dark for 10 h.

### Methods

#### Insect Samples, Total RNA Extraction and cDNA Synthesis

Male and female adults of 1, 5, 10, and 13 days (mated and unmated) after copulation were anesthetized with CO_2_ and different tissues such as antennae, wings, legs, abdomens, and heads were separated. Total RNA was extracted using TRIzol reagent (Invitrogen, Carlsbad, CA, United States) according to the manufacturer’s protocol, and the RNA concentration was determined with an ultraviolet spectrophotometer (Eppendorf BioPhotometer Plus, Germany), before reverse transcription. cDNA for qPCR was synthesized using PrimerScript RT Reagent kits with gDNA Eraser (Takara Bio, Otsu, Japan).

#### Temporal and Spatial Expression Profiles of MaltOBP9 and MaltOBP10

The qRT-PCR sample mixtures contained 10 μL of SYBR Premix Ex Taq II, 0.5 μL of each primer (10 μM), 2.5 μL of cDNA, and 6.5 μL sterilized ultrapure H_2_O. The qPCR conditions comprised an initial 3-min step at 95°C, followed by 40 cycles at 95°C for 10 s and 59°C for 30 s, and 81 cycles at 55°C for 6 s. The *M. alternatus* β-actin gene (NCBI accession number KX428475) was used as an internal control. Primer sequences were designed using Primer-BLAST via the NCBI website (Table 1). Data were analyzed using the comparative 2^–ΔΔ*CT*^ method (Thomas et al., 2008). Statistical analyses were performed with STATISTICA 7.

**TABLE 1:** List of primers used in this study.

Primer	Sequence
MaltOBP9-qPCR-F	5′-ATTTGGCACTGTGAATGCGG-3′
MaltOBP9-qPCR-R	5′-GAACGTGTCCGGCTATACCA-3′
MaltOBP10-qPCR-F	5′-ACCCACATGACGAAAAACTGC-3′
MaltOBP10-qPCR-R	5′-GCATTTCCTAAGAGCGGCGA-3′
β-actin-F	5′-CGCCCCATCCACCATGAAGA-3′
β-actin-R	5′-AGAGGGAGGCGAGGATGGAT-3′
MaltOBP9-clone-F	5′-GGAATTCATGAGAACTTGTGCTATAGTTGTTT-3′
MaltOBP9-clone-R	5′-CCGCTCGAGCTAATGATGGTGATGAACGTGTCCG-3′
MaltOBP10-clone-F	5′-GGAATTCATGCACAAGGCAGTTGTGAAAATGT-3′
MaltOBP10-clone-R	5′-CCGCTCGAGTTACACCAAAAACCAATTCTCCCTG-3′

### Cloning and Construction of Recombinant Plasmids

To clone the MaltOBP genes, cDNA was prepared from total RNA by reverse transcription using the RT-PCR (Reverse Transcription-Polymerase Chain Reaction system) (Promega) according to the manufacturer’s instructions. RT-PCR was performed using specific primer pairs (Table 1) with rTaq DNA polymerase (Takara Bio Inc., Shiga, Japan).

The PCR conditions comprised an initial 3-min step at 94°C, followed by 30 cycles at 94°C for 30 s, the specific melting temperature for 30 s, and 72°C for 1 min, before a final 10-min step at 72°C. The PCR products were confirmed by agarose gel electrophoresis and ligated into the pMD-18T vector using a 1:5 (plasmid:insert) molar ratio, before incubating for 0.5 h at 4°C. The ligation products were transformed into DH5α *Escherichia coli* competent cells and grown on lysogeny broth (LB) solid medium with 10 mg/mL ampicillin. Positive colonies were selected and grown in LB liquid medium with ampicillin, and then sequenced. The pMD-18T plasmids containing positive clones were digested with *EcoR*I and *Xho*I for 3 h at 37°C. The products were then separated on agarose gels. The target fragments were purified from the gels and ligated into the digested pET-20b plasmids, before the recombinant plasmids were transformed into DH5α *E. coli* competent cells and grown on LB solid medium with 10 mL ampicillin (10 mg/mL). Selected colonies were grown in LB liquid medium with ampicillin and sequenced. The correct recombinant plasmids were then transformed into BL21(DE3) pLySs *E. coli* competent cells. A single clone was identified and cultivated overnight in LB liquid medium with ampicillin on a shaker at 200 rpm and 37°C. The resulting plasmids were sequenced and it was confirmed that they encoded the mature proteins.

### Recombinant Protein Expression and Purification

A single positive clone was propagated overnight at 37°C in 5 mL of LB broth containing 50 μg/mL ampicillin. The culture was inoculated into 1 L of fresh medium and the bacteria were cultured for 2–3 h at 37°C until the medium reached an optical density of 0.6–0.8 at 600 nm. To induce the expression of the protein, isopropyl-β-D-thiogalactopyranoside (IPTG) was added at a final concentration of 0.1 mM in the culture medium and the bacteria were cultured for 3–4 h at 37°C. The cells were collected by centrifugation at 5,000 × *g* for 5 min and the cell pellet was then suspended in 50 mL of 50 mM Tris-HCl at pH 8.0. The suspension was sonicated on ice (the sonication process was divided into bursts with cooling between each treatment) and the lysate was centrifuged at 1,000 rpm for 1 h at 4°C.

The recombinant proteins were present as inclusion bodies. The lysate was centrifuged at 5,000 × *g* for 25 min at 4°C and the sediment was collected. To solubilize the recombinant proteins, the pellet obtained from each 1-L culture was dissolved in 10 mL of 8 M urea and 1 mM DTT (DL-Dithiothreitol) in 50 mM Tris buffer at pH 7.4, before diluting to 100 mL with Tris buffer and dialyzing three times against Tris buffer at pH 7.4. The proteins were purified in 50 mM Tris buffer at pH 7.4 using combinations of chromatographic steps on anion-exchange resins, i.e., DE-52 and QFF (GE-Healthcare, Beijing, China). The purity of the proteins was assessed by sodium dodecyl sulfate-polyacrylamide gel electrophoresis (SDS-PAGE).

### Scanning Electron Microscopy

Antennae from *M. alternatus* Hope adults of both sexes anesthetized with CO_2_ were dissected, kept overnight in a detergent solution, cleaned by ultrasound for 30 s, rinsed in water, and dehydrated with a graded ethanol series (5 min each in 30, 50, 70, 75, 80, 85, 90, 95, and 100% alcohol). Next, they were air dried and mounted on stubs with double-sided sticky tape. All of the specimens were gold coated using a Blazers SCD 040 sputter coater and subsequently observed with a scanning electron microscope (JSM-6390LV, NTC, Japan) at 20 kV.

### Antibody Preparation and Extraction of Tissue Proteins

Healthy adult rabbits were injected every 2 weeks subcutaneously and intramuscularly with the purified MaltOBP9 and MaltOBP10 proteins separately four times to produce polyclonal antibodies. Each OBP was emulsified with an equal volume of Freund’s complete adjuvant (Sigma, St Louis, MO, United States) (500 μg of purified protein) for the first injection, and incomplete adjuvant (300 μg each time) was added for the three subsequent injections. Seven days after the last injection, blood was collected to purify antibodies using a MAb Trap kit (GE Healthcare, Beijing, China). All operations were conducted according to ethical guidelines in order to minimize pain and discomfort for the animals. Antennae from 5-day-old adult males and females were ground to extract the total proteins, which were dissolved in 0.1 M phosphate-buffered saline (PBS) containing 10% phenylmethylsulfonyl fluoride (Boster, Wuhan, China) at pH 7.4. After a centrifugation at 5,000 × *g* for 30 min, the supernatant was collected to determine the concentration using a BCA Protein Quantification Kit (Yeasen, Shanghai, China).

### Immunoblotting

The purified proteins were used to prepare polyclonal antibodies in rabbits. The antennal extracts and purified proteins (15 μg) were separated by 15% SDS-PAGE and then transferred to polyvinylidene fluoride membranes (Millipore, Shanghai, China). Each membrane was blocked with 5% dry skimmed milk in 0.1 M PBS containing 0.1% Tween-20 at pH 7.4 for 2 h. The blocked membrane was incubated with the purified rabbit antibodies (diluted 1:4000) for 1 h, followed by horseradish peroxidase-conjugated goat anti-rabbit immunoglobulin G (diluted 1:5000) for 1 h. Three washes were conducted with PBS containing 0.1% Tween-20 between each process. Finally, the membrane was incubated with an Enhanced Chemiluminescence Western blot kit (CoWin Biotech, China) and the bands were checked with a ChemiDoc Imaging System (Bio-Rad, Hercules, CA, United States).

### Immunoelectron Microscopic Localization

Male and female antennae from 5-day-old *M. alternatus* adults were cut into small pieces and fixed overnight in 0.1 M PBS containing 4% paraformaldehyde and 2.5% glutaraldehyde at pH 7.4 and 4°C. Samples were dehydrated using an ethanol series and embedded in LR White resin (Taab, Aldermaston, Berks, United Kingdom). Ultrathin sections were cut with an LKB V Ultramicrotome (LKB Company, Bromma, Sweden) and mounted on formvar-coated 200 mesh grids. The sections were washed three times with 0.1 M PBS containing 50 mM glycine (buffer G) at pH 7.4, and blocked for 2 h at room temperature in 0.1 M PBS containing 2% gelatin, 5% bovine serum albumin, and 0.2% Tween-20 (buffer T) at pH 7.4. The sections were then stained overnight at 4°C with primary antibodies (dilution 1:4000) and subsequently stained with secondary antibodies (dilution 1:4000), which were coupled with 10-nm colloidal gold granules (Sigma, St Louis, MO, United States). After two washes in buffer T, buffer G, and water, the sections were finally examined with an H-7650 (Hitachi, Japan) transmission electron microscope. The preimmune serum from an uninjected healthy rabbit was used as a negative control.

### Fluorescence-Binding Assays

Fluorescence-binding assays were performed to determine the binding affinities of MaltOBP9 and MaltOBP10 with 17 volatiles in neutral and acidic pH conditions separately using *N*-phenyl-1-naphthylamine (1-NPN) as the fluorescent probe. 1-NPN and all of the other chemicals used in the binding assays were purchased from Sigma-Aldrich (St. Louis, MO, United States). All of the ligand stock solutions were prepared in spectrophotometric grade methanol. The binding constants for 1-NPN were determined by adding aliquots of 1 mM 1-NPN to separate samples containing 2 μM of each OBP in 50 mM Tris–HCl at pH 7.4 and room temperature. The binding of 1-NPN was measured at an excitation wavelength of 337 nm and the fluorescence intensity was recorded between 300 and 600 nm during a high-speed scan using an RF-5301PC fluorescence spectrophotometer with a 1-cm light path and a quartz cuvette. Saturation curves were constructed for the binding of 1-NPN with the OBPs and the dissociation constant (*K*_d_) of the binding reaction was calculated by performing Scatchard analysis of the data using the Prism 5 program (GraphPad, La Jolla, CA, United States). The binding analyses were performed based on the assumption that the protein was completely active and that the stoichiometry of binding was 1:1 at saturation. Aliquots of the competitor ligand were added to a sample containing 2 μM of each separate OBP and a standard concentration of 1-NPN. A decrease in the relative fluorescence intensity indicated that the competitor displaced 1-NPN from the binding site on the OBP. The binding data were collected during three independent high-speed scans. K_i_ representing the *K*_d_ of the competitor was determined based on the IC_50_ value (half maximal inhibitory concentration) for the competitor as the concentration of the competitor that displaced 50% of the bound 1-NPN at a standard total 1-NPN concentration (bound plus free) at equilibrium. K_i_ was calculated according to the following equation: K_i_ = [IC50]/(1+[1-NPN]/K), where 1-NPN is the free concentration of 1-NPN and K_1–NPN_ is K_d_ for the 1-NPN-OBP binding reaction determined by Scatchard analysis (Campanacci et al., 2001).

## Results

### MaltOBP9 and MaltOBP10 Gene Cloning

Based on the antennal transcriptome, specific primers were designed for MaltOBP9 and MaltOBP10, and applied in PCR (Supplementary Figure S1). The full open-reading frames were determined as 423 and 435 bp encoding 140 and 144 amino acid residues for MaltOBP9 and MaltOBP10, respectively. SignalP^1^ predicted signal peptides of 19 amino acid residues for MaltOBP9 and 25 amino acid residues for MaltOBP10. ExPASy^2^ predicted the isoelectric points and molecular weights of MaltOBP9 and MaltOBP10 as 6.43 and 15.4 kDa, and 5.05 and 16.9 kDa, respectively. The amino acid sequences of MaltOBP9 and MaltOBP10 were aligned with those of the corresponding OBPs from other species. BLAST analyses showed that MaltOBP9 has four Cys residues and it is a minus-C OBP whereas MaltOBP10 has six Cys residues and it is a classic OBP (Supplementary Figure S2).

### Expression Profiles of MaltOBP9 and MaltOBP10

**FIGURE 1:**
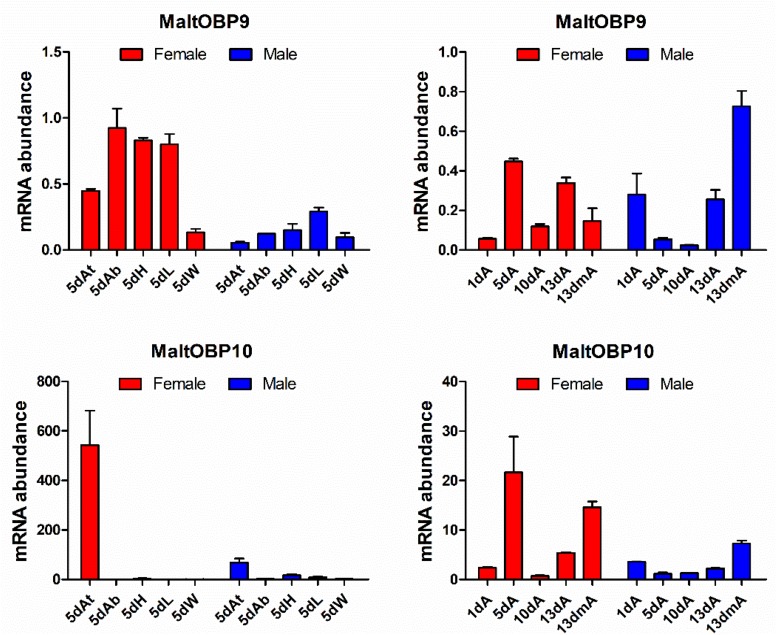
Expression profiles of MaltOBP9 and MaltOBP10. Relative transcript levels of MaltOBP9 and MaltOBP10 were analyzed in different tissues from 5-days-old adults and during different developmental stages. β-actin was used as internal control to normalize transcript levels in each sample. At/A, antennae; Ab, abdomens; H, head; L, leg; W, wing; m, mated.

The gene transcript levels of MaltOBP9 and MaltOBP10 were examined in the antennae during different developmental stages and in different tissues from 5-day-old males and females (Figure 1). MaltOBP9 was expressed in a broad range of different tissues, including the antenna, head, abdomen, leg, and wing, whereas MaltOBP10 was antenna-specific. In addition, the expression levels of the two genes did not exhibit obvious regular patterns in the antennae during different developmental stages.

### Types of Sensilla on the Antenna in *M. alternatus*

**FIGURE 2:**
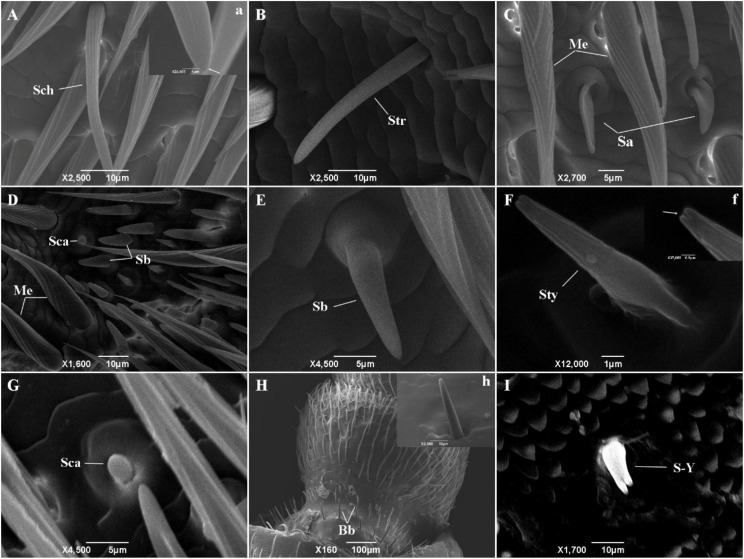
The types of sensilla present on *M. alternatus* antennae. Nine types of sensilla were found on *M. alternatus* antennae: Sch, Sensilla chaetica **(A)**; Str, Sensilla trichodea **(B)**; Me, Mechanoreceptor **(C,D)**; Sa, Sensilla auricillica **(C)**; Sca, sensilla campaniformia **(D,G)**; Sb, Sensilla basiconica **(D,E)**; Sty, Sensilla styloconica **(F)**; Bb, Böhm bristles **(H)**; S-Y, Y-shaped bifid sensilla **(I)**. White arrows in a and f showing pores at higher magnification.

At least nine types of sensilla were identified by scanning electron microscopy (Figure 2). Sensilla chaetica (Figure 2A, a) were inserted into articulatory sockets with blunt tips, where they had longitudinal grooves on the wall and tapered toward the tip. They were slightly curved to strongly curved, and terminal pores were present on the apex. Sensilla trichodea (Figure 2B) emerged out of articulatory sockets and stood outside the integument forming an angle of 60–90°. They were smooth-walled and blunt-tipped, and thinner and longer than sensilla basiconica. Mechanoreceptors (Figures 2C,D) were the most abundant sensilla on the antennae, where they were positioned relatively parallel to the body of the antenna. Their walls were densely grooved and formed an inverted V-type from the apex to the base. They had a stout base and sharp points, and were always bent near the top. Sensilla basiconica (Figures 2D,E) were located on a characteristic dome at a slant and they pointed toward the end of the antenna. They were blunt-tipped and smooth-walled pegs without grooves. In general, they emerged in clusters and stood straight, although some were slightly curved near the base. Sensilla auricillica (Figure 2C) resembled the ears or leaves of graminaceous plants and they were located in bulging pedestals, which were moderately concave and indented in the center. Obvious depressions were visible on the surface but they had no grooves on the cuticular wall. Sensilla styloconica (Figure 2F, f) were blunt-tipped pegs and they possessed longitudinal grooves distally. They were typically inserted into a small dome and had no articulating socket, and they were oriented perpendicular to the antennal surface with openings at the peak. Sensilla campaniformia (Figures 2D,G) were small mastoid processes resembling bell jars, where their form was cylindrical and thick-set. Böhm bristles (Figure 2H, h) were only present on the ball joint of the scape and at the base of the pedicle. They were needle-shaped and emerged from a raised base with an articulatory socket. Y-shaped bifid sensilla (Figure 2I) were found in low and variable densities in only a few samples. They were inserted into a deep socket and characterized by a Y-shaped bifurcation near the tip.

### Specific Expression of MaltOBP9 and MaltOBP10 in Antennal Sensilla

**FIGURE 3:**
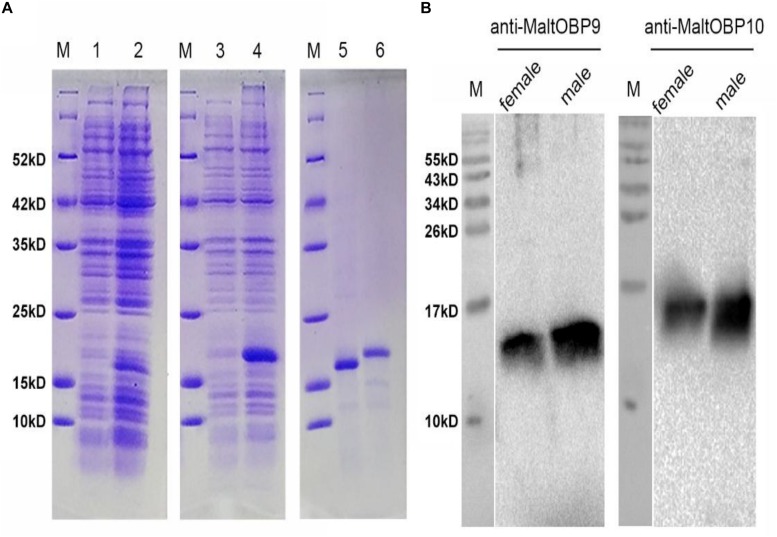
SDS-PAGE analyses of recombinant OBPs and Western blot analysis of MaltOBP9 and MaltOBP10 expression in total protein extracts of male and female antennae of *M. alternatus*. **(A)** SDS-PAGE analyses showing the expression and purification of the recombinant OBPs. M, molecular marker. Lane 1 and 3 represented the bacterial cells before induction by IPTG. Lane 2 and 4 represented the bacterial cells which express recombinant OBPs of MaltOBP9 and MaltOBP10 respectively after induction by IPTG. Lane 5 and 6 represented purified recombinant MaltOBP9 and MaltOBP10 respectively. **(B)** The cross-reaction of anti-MaltOBP9 and anti- MaltOBP10 with total protein extracts of male and female antennae of *M. alternatus* respectively. A clear single target band indicates that the obtained antibody with high specificity can be used for further experiments. MaltOBP9 and MaltOBP10 existed in the total protein extracts of male and female antennae.

**FIGURE 4:**
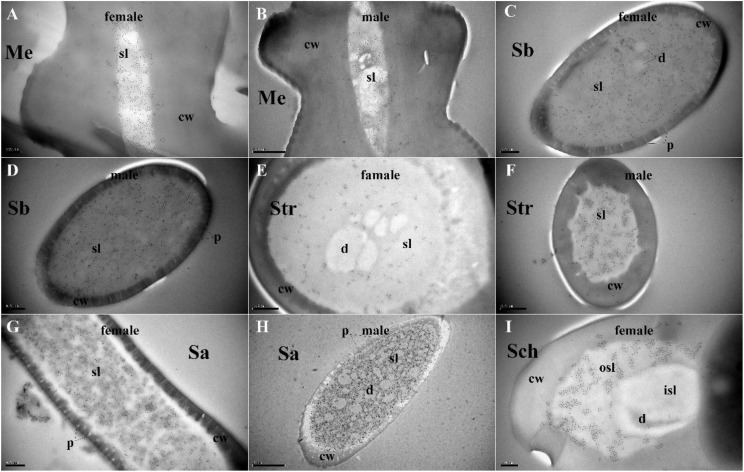
Immunocytochemical localization of MaltOBP9 in different sensilla of female and male *M. alternatus* antennae. Anti-MaltOBP9 labeled by gold granules were observed in the sensillum lymph of four different sensilla on female and male *M. alternatus* antennae: Mechanoreceptor **(A,B)**; Sensilla basiconica **(C,D)**; Sensilla trichodea **(E,F)**; Sensilla auricillica **(G,H)**; Sensilla chaetica **(I)**. Few grains found in over the cuticle and the dendrites represent non-specific background. Notably, mechanoreceptor, the walls of which were densely grooved and make inverted V type from apex to the base, had no neuronal dendrites reside. Meanwhile, the outer sensillum lymph, but not the inner sensillum lymph, of the Sensilla chaetica showed strong staining by Anti-MaltOBP9. Abbreviations: cw, cuticular wall; sl, sensillum lymph; isl, inner sensillum lymph; osl, outer sensillum lymph; p, pore; d, dendrities.

**FIGURE 5:**
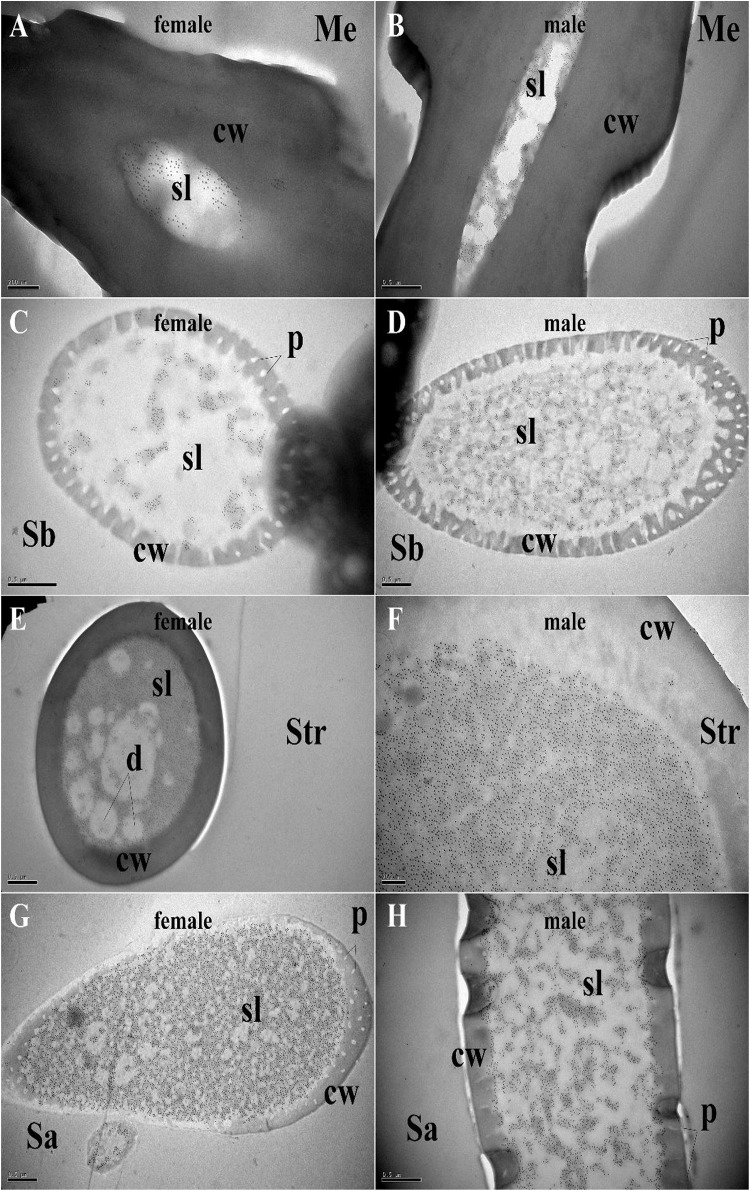
Immunocytochemical localization of MaltOBP10 in different sensilla of female and male *M. alternatus* antennae. Anti-MaltOBP10 labeled by gold granules were also observed in the sensillum lymph of four different sensilla on female and male *M. alternatus* antennae: Mechanoreceptor **(A,B)**; Sensilla basiconica **(C,D)**; Sensilla trichodea **(E,F)**; Sensilla chaetica **(G,H)**. No staining was observed in the sensillum lymph of the Sensilla chaetica. cw, cuticular wall; sl, sensillum lymph; isl, inner sensillum lymph; osl, outer sensillum lymph; p, pore; d, dendrities.

Polyclonal antibodies were prepared using the purified proteins (Figure 3A). Western blot examinations were conducted to check the specificity of the antibodies The two clear single target bands indicated that both anti-MaltOBP9 and anti-MaltOBP10 recognized their targets among the total proteins from the male and female antenna (Figure 3B). The polyclonal antibodies were then used to localize MaltOBP9 and MaltOBP10 in the antennal sensilla from female and male *M. alternatus*. The sensillum lymph was labeled in these sensilla, whereas the cuticular wall and dendrites were not labeled. The immunocytochemistry results showed that mechanoreceptors, sensilla basiconica, sensilla trichodea, and sensilla auricillica on the antennae from females and males were all labeled with both anti-MaltOBP9 and anti-MaltOBP10 (Figures 4, 5). In female antennae, anti-MaltOBP9 labeled with gold granules were observed in the outer sensillum lymph of the sensilla chaetica (Figure 4I). However, it is difficult for us to affirm the immunocytochemistry results in male Sch according to the obtained images (not shown).

### Fluorescence-Binding Assays

**FIGURE 6:**
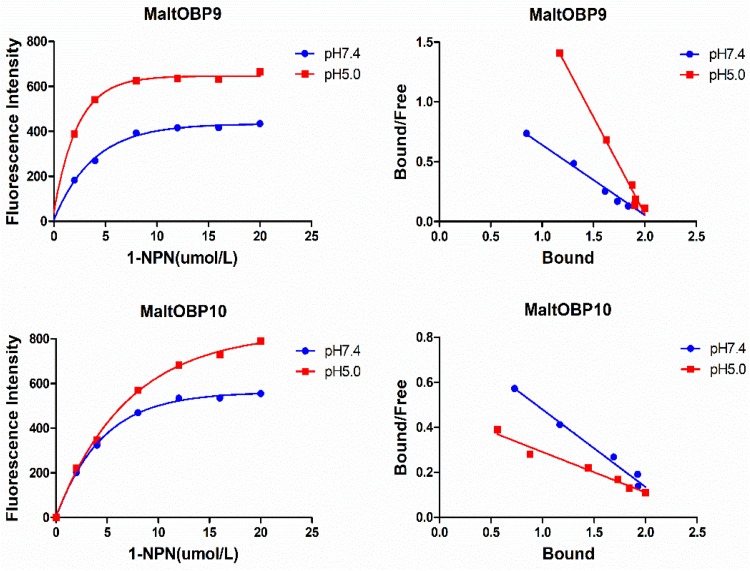
The binding of fluorescent probe 1-NPN with MaltOBP9 and MaltOBP10. Binding curve and Scatchard plot of 1-NPN binding to MaltOBP9 and MaltOBP10 in higher pH (7.4) and lower pH (5.0) are shown by the blue and red lines, respectively. The binding curves were gradually saturated with increasing 1-NPN concentrations and the Scatchard plot shown a straight line indicated that the bindings of 1-NPN were saturable and consistent with a single population of binding sites.

**FIGURE 7:**
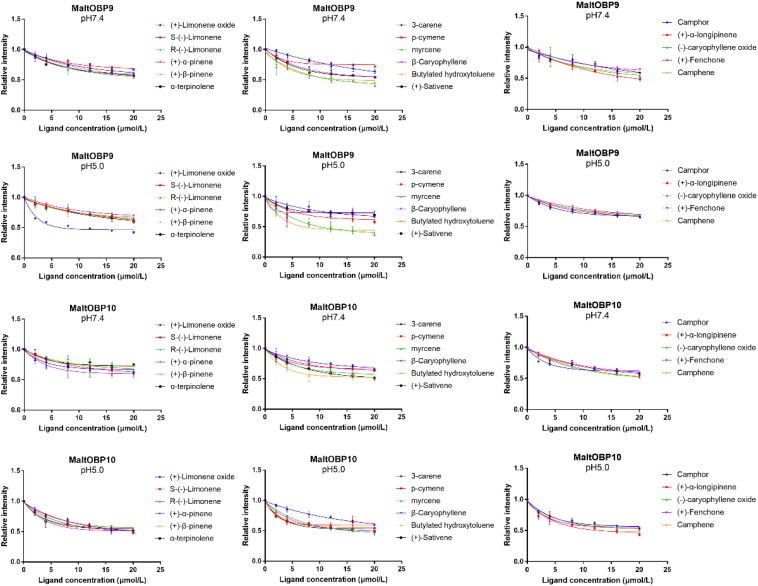
Competitive binding curves of MaltOBP9-1-NPN and MaltOBP10-1-NPN complexes to ligands.

After confirmation by SDS-PAGE, the purified MaltOBP9 and MaltOBP10 proteins were used in fluorescence-binding assays to determine their binding affinities with different volatiles that come from forest. First, we analyzed the binding of the fluorescent probe 1-NPN with MaltOBP9 and MaltOBP10 (Figure 6). The binding between 1-NPN and the proteins gradually saturated as the 1-NPN concentration increased and the Scatchard plot comprised a straight line, which indicated that the binding of 1-NPN was saturable and consistent with a single population of binding sites (Figure 6). The binding affinities of 18 potential competitor volatiles (including volatiles from pine bark) with the proteins are shown in Table 2, and the competitive binding curves are shown in Figure 7. The binding affinities (K_i_ < 50) (Zheng et al., 2016) with all of the ligands indicted the broad binding ranges of MaltOBP9 and MaltOBP10 at pH 7.4. As for the binding affinities measured at pH 5.0, MaltOBP9 exhibited some differences: several ligands such as *p*-cymene had lower binding affinities and some of the binding affinities were even lost (K_i_ > 50), but several ligands such as (+)-β-pinene were not affected and ligands such as myrcene had slightly higher binding affinities. The change in the pH appeared to have less impact on the binding with MaltOBP10. Most of the ligands exhibited lower binding affinities but they maintained their high binding abilities with MaltOBP10.

**TABLE 2:** Binding data [indicated by K_i_ (μM)] of MaltOBP9 and MaltOBP10 with different ligands.

Ligand	Purity (%)	MaltOBP9		MaltOBP10	
		pH7.4	pH5.0	pH7.4	pH5.0
1-NPN	≥98	1.70 ± 0.21	0.62 ± 0.18	1.35 ± 0.09	5.88 ± 0.69
(+)-Limonene oxide	≥97	43.34 ± 0.74	48.75 ± 4.51	19.78 ± 1.78	46.07 ± 4.9
S-(−)-Limonene	≥99	29.19 ± 1.81	11.96 ± 0.86	18.36 ± 0.67	51.7 ± 5.74
R-(+)-Limonene	≥97	17.1 ± 2.24	>50	23.92 ± 1.89	55.48 ± 9.88
(+)-α-pinene	≥99	21.1 ± 2.84	>50	13.29 ± 1.94	17.29 ± 3.16
(+)-β-pinene	≥99	21.04 ± 0.92	21.68 ± 0.65	25.77 ± 3.78	62.78 ± 1.08
α-terpinolene	≥85	22.21 ± 0.34	18.46 ± 0.23	17.5 ± 0.33	15.55 ± 1.44
3-carene	≥90	25.15 ± 0.76	38.12 ± 0.23	42.53 ± 0.82	32.55 ± 4.28
*p*-cymene	≥99	11.73 ± 0.23	>50	23.79 ± 2.33	35.44 ± 0.63
Myrcene	≥90	7.77 ± 0.14	3.96 ± 0.78	12.37 ± 0.5	19.37 ± 0.02
β-Caryophyllene	≥98.5	16.365 ± 0.04	129.47 ± 9.52	16.34 ± 1.97	23.48 ± 1.72
Butylated hydroxytoluene	≥98	9.58 ± 0.28	3.57 ± 0.58	20.09 ± 2.75	9.51 ± 0.44
(+)-Sativene	≥98	19.47 ± 0.36	>50	16.51 ± 1.42	12.75 ± 1.21
Camphor	≥95	25.88 ± 0.76	50.51 ± 3.68	26.21 ± 1.76	21.19 ± 3.49
(+)-α-longipinene	≥99	12.51 ± 0.67	34.67 ± 0.94	11.62 ± 0.74	21.25 ± 1.75
(−)-caryophyllene oxide	≥99	15.26 ± 0.94	39.28 ± 0.73	16.37 ± 0.4	16.68 ± 1.27
(+)-Fenchone	≥95	27.75 ± 1.04	46.16 ± 2.59	22.72 ± 1.23	24.71 ± 1.29
Camphene	≥95	22.57 ± 1.08	>50	20.58 ± 1.22	14.8 ± 0.57

## Discussion

In this study, we cloned and identified MaltOBP9 and MaltOBP10 from *M. alternatus* Hope, which were assigned to the minus-C OBP and classic insect OBP subfamilies, respectively. Previous studies have confirmed the olfactory functions of OBPs as binding and transport odorant molecules, and the majority are expressed in the antennae (Pelosi et al., 2006). Some OBPs are only highly expressed in males or females, and they seem to have roles in pheromone perception or other special functions (Mastrobuoni et al., 2013; Ma et al., 2016). MaltOBP10 was highly expressed in the female antennae, which suggests that this OBP may be involved in female-specific olfactory behavior. Similar to MaltOBP9 in our study, many other OBPs have been found in non-sensory organs such as legs and wings (Li et al., 2005; Forêt and Maleszka, 2006; Pitts et al., 2011). In the honey bee *Apis mellifera*, only nine out of twenty-one OBPs are antenna specific (Forêt and Maleszka, 2006). Considering the hydrophobic binding cavities in the centers of the proteins, which are structural characteristics of the OBP family, the transportation of hydrophobic molecules in different tissues may be a common feature of OBPs, but OBPs are not restricted to olfaction and they are likely to be involved in a broad range of physiological functions (Forêt and Maleszka, 2006; Brito et al., 2016).

Various hair-like sensilla located on the antennae are involved in multiple sensory modalities, such as olfaction, gustation, mechanoreception, thermoreception, and hygroreception (Altner and Prillinger, 1980; Zacharuk, 1985). A previous study found six types of antennal sensilla in *M. alternatus* (Sun et al., 2010), and we identified three additional types of sensilla, as follows. Böhm bristles correspond to “aporous Böhm’s sensilla” in *Phoracantha recurva* and they may be mechanoreceptors involved in proprioception (Faucheux, 2011). Sensilla campaniformia are the same as those found in *Paussus favieri* (Di et al., 2012) and they are known to respond to variations in temperature and humidity (Meiners et al., 2003; Must et al., 2006, 2010; Merivee et al., 2010). In addition, Y-shaped bifid sensilla were identified in *Rhynchophorus palmarum* (Saïd et al., 2003) and *Aphidius rhopalosiphi* (Bourdais et al., 2006). Morphological alterations due to stress during development might explain their presence and they may not be a distinct sensillar type (Mackay et al., 2014). According to the structural features of the antennal sensilla in *M. alternatus*, they were classified into three categories: mechanoreceptors that are not chemical sensilla; sensilla basiconica, sensilla trichodea, and sensilla auricillica as olfactory sensilla; and sensilla chaetica and sensilla styloconica as possible gustatory sensilla (Sun et al., 2010). Our immunocytochemistry results indicated the high expression of MaltOBP9 and MaltOBP10 in the sensillum lymph of sensilla basiconica, sensilla trichodea, sensilla auricillica, and sensilla chaetica, thereby providing further evidence for their participation in semiochemical detection. These results are consistent with those obtained in other species based on *in situ* hybridization or immunocytochemistry experiments (Steinbrecht et al., 1995; Forstner et al., 2006; Gu et al., 2011a; Zhang et al., 2011; Sun et al., 2014). Moreover, these two proteins were located in the inner cavity of the mechanoreceptor, so we suggest that these proteins might participate in other sensory processes or that the mechanoreceptors may participate in chemoreception in an unknown manner.

Previous studies have reported the specific binding of ligands with OBPs, such as the pheromone bombykol [(10E,12Z)-hexadeca-10,12-dien-1-ol] with *B. mori* BmorPBP1 (Sandler et al., 2000). However, most of these studies indicated that OBPs often have a broad ligand-binding affinity, and they can bind with a wide range of ligands according to fluorescence-binding assays (Yin et al., 2012; Jin et al., 2014; Brito et al., 2016). The abilities of MaltOBP9 and MaltOBP10 to bind with a wide variety of ligands were determined in our study. MaltOBP9 and MaltOBP10 had a broad range of binding abilities even in an acid environment. Considering the immunocytochemical localization of MaltOBP9 in various sensilla and its expression in a broad range of different tissues, MaltOBP9 may play a role in binding with a broad range of ligands in many physiological pathways, where non-sensory organs are even involved in olfactory recognition. MaltOBP10 is specifically expressed in the antenna of females and it may play a role in the fundamental olfactory recognition process. Fluorescence-binding assays have some shortcomings, for instance, high binding affinities showed by this experiment do not always mean the real binding between the OBP and the ligand, in some cases, that is the consequence of molecular collisions (Yang et al., 2017). Thus more information about the binding characteristics of OBPs, such as quenching analysis and secondary structural transformation, should be obtained in further experiments.

## Data Availability Statement

The raw data supporting the conclusions of this manuscript will be made available by the authors, without undue reservation, to any qualified researcher.

## Author Contributions

D-ZL and M-QW designed the study. X-FH and R-NY collected the data. D-ZL, X-FH, and R-NY conducted the statistical analyses. D-ZL, J-YC, and M-QW wrote the manuscript with input and revisions from all authors.

## Conflict of Interest

The authors declare that the research was conducted in the absence of any commercial or financial relationships that could be construed as a potential conflict of interest.

## Footnotes

1 http://www.cbs.dtu.dk/services/SignalP/

2 https://web.expasy.org/compute_pi/

## References

[B1] Altner H. Prillinger L. (1980). Ultrastructure of invertebrate chemo-, thermo-, and hygroreceptors and its functional significance. *Int. Rev. Cytol.* 67 69–139. 10.1016/s0074-7696(08)62427-4

[B2] Benton R. Vannice K. S. Vosshall L. B. (2007). An essential role for a CD36-related receptor in pheromone detection in *Drosophila*. *Nature* 450 289–293. 10.1038/nature06328 17943085

[B3] Bourdais D. Vernon P. Krespi L. Lannic J. L. Baaren J. V. (2006). Antennal structure of male and female *Aphidius* rhopalosiphi DeStefani-Peres (*Hymenoptera*:*Braconidae*): description and morphological alterations after cold storage or heat exposure. *Microsc. Res. Tech.* 69 1005. 10.1002/jemt.20378 17019677

[B4] Brito N. F. Moreira M. F. Melo A. C. (2016). A look inside odorant-binding proteins in insect chemoreception. *J. Insect Physiol.* 95 51–65. 10.1016/j.jinsphys.2016.09.008 27639942

[B5] Campanacci V. Krieger J. Bette S. Sturgis J. N. Lartigue A. Breer H. (2001). Revisiting the specificity of *Mamestra brassicae* and *Antheraea polyphemus* pheromone-binding proteins with a fluorescence binding assay. *J. Biol. Chem.* 276 20078–20084. 10.1074/jbc.M100713200 11274212

[B6] Damberger F. F. Ishida Y. Leal W. S. Wüthrich K. (2007). Structural basis of ligand binding and release in insect pheromone-binding proteins: NMR structure of *Antheraea* polyphemus PBP1 at pH 4.5. *J. Mol. Bio.* 373 811–819. 10.1016/j.jmb.2007.07.078 17884092

[B7] De Biasio F. Riviello L. Bruno D. Grimaldi A. Congiu T. Sun Y. F. (2015). Expression pattern analysis of odorant-binding proteins in the pea aphid *Acyrthosiphon pisum*. *Insect Sci.* 22 220–234. 10.1111/1744-7917.12118 24591440

[B8] Di G. A. Maurizi E. Stacconi M. V. Romani R. (2012). Functional structure of antennal sensilla in the myrmecophilous beetle *Paussus favieri* (*Coleoptera*, *Carabidae*, *Paussini*). *Micron* 43 705–719. 10.1016/j.micron.2011.10.013 22365951

[B9] Fan J. Kang L. Sun J. (2007). Role of host volatiles in mate location by the Japanese pine sawyer, *Monochamus alternatus* hope (*Coleoptera*: *Cerambycidae*). *Environ. Entomol.* 36 58 10.1603/0046-225x(2007)36[58:rohvim]2.0.co;2 17349117

[B10] Faucheux M. J. (2011). Antennal sensilla of the yellow longicorn beetle Phoracantha recurva Newman, 1840: distribution and comparison with *Phoracantha semipunctata* (*Fabricius*). *Bull. Inst. Sci.* 33 19–29.

[B11] Forêt S. Maleszka R. (2006). Function and evolution of a gene family encoding odorant binding-like proteins in a social insect, the honey bee (*Apis mellifera*). *Genome Res.* 16 1404–1413. 10.1101/gr.5075706 17065610 PMC1626642

[B12] Forstner M. Gohl T. Breer H. Krieger J. (2006). Candidate pheromone binding proteins of the silkmoth Bombyx mori^∗^. *Invert. Neurosci.* 6 177–187. 10.1007/s10158-006-0032-0 17082917

[B13] Gao X. Wang M. Q. (2015). A cDNA library from the antenna of *Monochamus alternatus* Hope and binding properties of odorant-binding proteins. *J. Appl. Entomol.* 139 229–236. 10.1111/jen.12136

[B14] Gu S. H. Wang W. X. Wang G. R. Zhang X. Y. Guo Y. Y. Zhang Z. (2011a). Functional characterization and immunolocalization of odorant binding protein 1 in the lucerne plant bug, *Adelphocoris lineolatus* (GOEZE). *Arch. Insect Biochem. Physiol.* 77 81–99. 10.1002/arch.20427 21541988

[B15] Gu S. H. Wang S. P. Zhang X. Y. Wu K. M. Guo Y. Y. Zhou J. J. (2011b). Identification and tissue distribution of odorant binding protein genes in the lucerne plant bug *Adelphocoris lineolatus* (Goeze). *Insect Biochem. Mol. Biol.* 41 254–263. 10.1016/j.ibmb.2011.01.002 21232599

[B16] He P. Zhang J. Liu N. Y. Zhang Y. N. Yang K. Dong S. L. (2011). Distinct expression profiles and different functions of odorant binding proteins in nilaparvata lugens stål. *Plos One* 6:e28921. 10.1371/journal.pone.0028921 22174925 PMC3235172

[B17] He X. He Z. B. Zhang Y. J. Zhou Y. Xian P. J. Qiao L. (2016). Genome-wide identification and characterization of odorant-binding protein (OBP) genes in the malaria vector *Anopheles* sinensis (*Diptera*: *Culicidae*). *Insect Sci.* 23 366–376. 10.1111/1744-7917.12333 26970073

[B18] Jayanthi K. P. Kempraj V. Aurade R. M. Roy T. K. Shivashankara K. S. Verghese A. (2014). Computational reverse chemical ecology: virtual screening and predicting behaviorally active semiochemicals for *Bactrocera dorsalis*. *Bmc Genomics* 15:209. 10.1186/1471-2164-15-209 24640964 PMC4003815

[B19] Jeong Y. T. Shim J. Oh S. R. Yoon H. I. Kim C. H. Moon S. J. (2013). An Odorant-binding protein required for suppression of sweet taste by bitter chemicals. *Neuron* 79 725. 10.1016/j.neuron.2013.06.025 23972598 PMC3753695

[B20] Jin J. Y. Li Z. Q. Zhang Y. N. Liu N. Y. Dong S. L. (2014). Different roles suggested by sex-biased expression and pheromone binding affinity among three pheromone binding proteins in the pink rice borer, *Sesamia inferens* (Walker) (*Lepidoptera*: *Noctuidae*). *J. Insect Physiol.* 66 71–79. 10.1016/j.jinsphys.2014.05.013 24862154

[B21] Jin X. Ha T. S. Smith D. P. (2008). Snmp is a signaling component required for pheromone sensitivity in *Drosophila*. *Proc. Natl. Acad. Sci. U. S. A.* 105 10996. 10.1073/pnas.0803309105 18653762 PMC2504837

[B22] Kobayashi F. Yamane A. Ikeda T. (1984). The Japanese pine sawyer beetle as the vector of pine wilt disease. *Annu. Rev. Entomol.* 29 115–135. 10.1146/annurev.en.29.010184.000555

[B23] Krieger J. Von N. E. Mameli M. Pelosi P. Breer H. (1996). Binding proteins from the antennae of *Bombyx mori*. *Insect Biochem. Mol. Biol.* 26 297 10.1016/0965-1748(95)00096-8 8900598

[B24] Laughlin J. D. Ha T. S. Jones D. N. Smith D. P. (2008). Activation of pheromone-sensitive neurons is mediated by conformational activation of pheromone-binding protein. *Cell* 133 1255–1265. 10.1016/j.cell.2008.04.046 18585358 PMC4397981

[B25] Leal W. S. (2013). Odorant reception in insects: roles of receptors, binding proteins, and degrading enzymes. *Annu. Rev. Entomol.* 58 373. 10.1146/annurev-ento-120811-153635 23020622

[B26] Leite N. R. Krogh R. Xu W. Ishida Y. Iulek J. Leal W. S. (2011). Structure of an odorant-binding protein from the mosquito *Aedes aegypti* suggests a binding pocket covered by a pH-sensitive “Lid”. *Plos One* 4:e8006. 10.1371/journal.pone.0008006 19956631 PMC2778553

[B27] Lescop E. Briand L. Pernollet J. C. Guittet E. (2009). Structural basis of the broad specificity of a general odorant-binding protein from honeybee. *Biochemistry* 48 2431–2441. 10.1021/bi802300k 19186989

[B28] Li D. Z. Yu G. Q. Yi S. C. Zhang Y. Kong D. X. Wang M. Q. (2015). Structure-based analysis of the ligand-binding mechanism for DhelOBP21, a C-minus odorant binding protein, from *Dastarcus helophoroides* (*Fairmaire*; *Coleoptera*: *Bothrideridae*). *Int. J. Biol. Sci.* 11 1281–1295. 10.7150/ijbs.12528 26435694 PMC4582152

[B29] Li H. Zhang G. Wang M. (2012). Chemosensory protein genes of batocera horsfieldi (hope): identification and expression pattern. *J. Appl. Entomol.* 136 781–792. 10.1111/j.1439-0418.2012.01712.x

[B30] Li N. Sun X. Wang M. Q. (2017). Expression pattern and ligand-binding properties of odorant-binding protein 13 from *Monochamus alternatus* hope. *J. Appl. Entomol.* 141 751–757. 10.1111/jen.12396

[B31] Li Z. X. Pickett JAField L. M. Zhou J. J. (2005). Identification and expression of odorant-binding proteins of the malaria-carrying mosquitoes *Anopheles gambiae* and *Anopheles arabiensis*. *Arch. Insect Biochem. Physiol.* 58 175–189. 15717318 10.1002/arch.20047

[B32] Lopes O. Barata E. N. Mustaparta H. Araujo J. (2002). Fine structure of antennal sensilla basiconica and their detection of plant volatiles in the eucalyptus woodborer, *Phoracantha semipunctata* Fabricius (*Coleoptera*: *Cerambycidae*). *Arthropod Struct. Dev.* 31 1–13. 10.1016/s1467-8039(02)00011-7 18088966

[B33] Ma M. Chang M. M. Lei C. L. Yang F. L. (2016). A garlic substance disrupts odorant-binding protein recognition of insect pheromones released from adults of the angoumois grain moth. *Sitotroga cerealella* (*Lepidoptera*: *Gelechiidae*). *Insect Mol. Biol.* 25 530. 10.1111/imb.12240 27111111

[B34] Mackay C. A. Sweeney J. D. Hillier N. K. (2014). Morphology of antennal sensilla of the brown spruce longhorn beetle, *Tetropium fuscum* (Fabr.) *(Coleoptera*: *Cerambycidae*). *Arthropod Struct. Dev.* 43 469. 10.1016/j.asd.2014.04.005 24768726

[B35] Maïbèche-Coisne M. Sobrio F. Delaunay T. Lettere M. Dubroca J. Jacquin-Joly E. (1997). Pheromone binding proteins of the moth *Mamestra brassicae* : specificity of ligand binding. *Insect Biochem. Mol. Biol.* 27 213–221. 10.1016/s0965-1748(96)00088-4

[B36] Mao Y. Xu X. Xu W. Ishida Y. Leal W. S. Ames J. B. (2010). Crystal and solution structures of an odorant-binding protein from the southern house mosquito complexed with an oviposition pheromone. *Proc. Natl. Acad. Sci. U.S.A.* 107 19102. 10.1073/pnas.1012274107 20956299 PMC2973904

[B37] Mastrobuoni G. Qiao H. Iovinella I. Sagona S. Niccolini A. Boscaro F. (2013). A proteomic investigation of soluble olfactory proteins in *Anopheles gambiae*. *Plos One* 8:e75162. 10.1371/journal.pone.0075162 24282496 PMC3839933

[B38] Meiners T. Wäckers F. Lewis W. J. (2003). Associative learning of complex odours in parasitoid host location. *Chem. Senses* 28 231 10.1093/chemse/28.3.231 12714445

[B39] Merivee E. Must A. Luik A. Williams I. (2010). Electrophysiological identification of hygroreceptor neurons from the antennal dome-shaped sensilla in the ground beetle Pterostichus oblongopunctatus. *J. Insect Physiol.* 56 1671–1678. 10.1016/j.jinsphys.2010.06.017 20615410

[B40] Must A. Merivee E. Luik A. Mänd M. Heidemaa M. (2006). Responses of antennal campaniform sensilla to rapid temperature changes in ground beetles of the tribe platynini with different habitat preferences and daily activity rhythms. *J. Physiol.* 52 506–513. 10.1016/j.jinsphys.2006.01.010 16527304

[B41] Must A. Merivee E. Luik A. Williams I. Ploomi A. Heidemaa M. (2010). Spike bursts generated by the thermosensitive (cold) neuron from the antennal campaniform sensilla of the ground beetle Platynus assimilis. *J. Insect Physiol.* 56 412–421. 10.1016/j.jinsphys.2009.11.017 19945461

[B42] Pelosi P. Zhou J. J. Ban L. P. Calvello M. (2006). Soluble proteins in insect chemical communication. *Cell. Mol. Life Sci.* 63 1658–1676. 10.1007/s00018-005-5607-0 16786224 PMC11136032

[B43] Pitts R. J. Rinker D. C. Jones P. L. Rokas A. Zwiebel L. J. (2011). Transcriptome profiling of chemosensory appendages in the malaria vector *Anopheles gambiae* reveals tissue-and sex-specific signatures of odor coding. *BMC Genomics* 12:271. 10.1186/1471-2164-12-27 21619637 PMC3126782

[B44] Saïd I. Tauban D. Renou M. Mori K. Rochat D. (2003). Structure and function of the antennal sensilla of the palm weevil *Rhynchophorus palmarum* (*Coleoptera*, *Curculionidae*). *J. Insect Physiol.* 49 857–872. 10.1016/s0022-1910(03)00137-9 16256688

[B45] Sandler B. H. Nikonova L. Leal W. S. Clardy J. (2000). Sexual attraction in the silkworm moth: structure of the pheromone-binding-protein–bombykol complex. *Chem. Biol.* 7 143–151. 10.1016/s1074-5521(00)00078-8 10662696

[B46] Spinelli S. Lagarde A. Iovinella I. Legrand P. Tegoni M. Pelosi P. (2012). Crystal structure of Apis mellifera OBP14, a C-minus odorant-binding protein, and its complexes with odorant molecules. *Insect Biochem. Mol. Biol.* 42 41. 10.1016/j.ibmb.2011.10.005 22075131

[B47] Steinbrecht R. A. Laue M. Ziegelberger G. (1995). Immunolocalization of pheromone-binding protein and general odorant-binding protein in olfactory sensilla of the silk moths Antheraea and Bombyx. *Cell Tissue Res.* 282 203–217. 10.1007/s004410050473

[B48] Sun F. Li X. R. Liu X. X. Zhang Q. W. (2010). Ultrastructure of six types of antennal sensilla in *Monochamus alternatus*. *Chin. Bull. Entomol.* 47 347–354.

[B49] Sun L. Xiao H. J. Gu S. H. Zhou J. J. Guo Y. Y. Liu Z. W. (2014). The antenna-specific odorant-binding protein AlinOBP13 of the alfalfa plant bug *Adelphocoris* lineolatus is expressed specifically in basiconic sensilla and has high binding affinity to terpenoids. *Insect Mol. Biol.* 23 417–434. 10.1111/imb.12089 24576076

[B50] Thomas S. Kenneth D. Livak J. (2008). Analyzing real-time PCR data by comparative CT method. *Nat. Protoc.* 3 1101–1108. 10.1038/nprot.2008.73 18546601

[B51] Tsitsanou K. E. Thireou T. Drakou C. E. Koussis K. Keramioti M. V. Leonidas D. D. (2012). *Anopheles gambiae* odorant binding protein crystal complex with the synthetic repellent DEET: implications for structure-based design of novel mosquito repellents. *Cellu. Mol. Life Sci.* 69 283–297. 10.1007/s00018-011-0745-z 21671117 PMC11114729

[B52] Venthur H. Mutis A. Zhou J. Quiroz A. (2015). Ligand binding and homology modelling of insect odorant-binding proteins. *Physiol. Entomol.* 39 183–198. 10.1111/phen.12066

[B53] Wang J. Li D.-Z. Min S.-F. Mi F. Zhou S.-S. Wang M.-Q. (2014). Analysis of chemosensory gene families in the beetle *Monochamus* alternatus and its parasitoid Dastarcus helophoroides. *Comp. Biochem. Physiol. Part D Genomics Proteomics* 11 1–8. 10.1016/j.cbd.2014.05.001 24893337

[B54] Wang L. Bi Y. D. Liu M. Li W. Liu M. Di S. F. (2019). Identification and expression profiles analysis of odorant-binding proteins in soybean aphid, *Aphis glycines* (*Hemiptera*: *Aphididae*). *Insect Sci.* 1–12. 10.1111/1744-7917.12709 31271503

[B55] Wei J. R. Yang Z. Q. Poland T. M. Du J. W. (2009). Parasitism and olfactory responses of *Dastarcus helophoroides* (*Coleoptera*: *Bothrideridae*) to different Cerambycid hosts. *Biocontrol* 54 733–742. 10.1007/s10526-009-9224-y

[B56] Wogulis M. Morgan T. Ishida Y. Leal W. S. Wilson D. K. (2006). The crystal structure of an odorant binding protein from *Anopheles gambiae*: evidence for a common ligand release mechanism. *Biochem. Biophys. Res. Commun.* 339 157–164. 10.1016/j.bbrc.2005.10.191 16300742

[B57] Yang R. N. Li D. Z. Yu G. Yi S. C. Zhang Y. Kong D. X. (2017). Structural transformation detection contributes to screening of behaviorally active compounds: dynamic binding process analysis of DhelOBP21 from *Dastarcus helophoroides*. *J. Chem. Ecol.* 43 1–13. 29063475 10.1007/s10886-017-0897-x

[B58] Yin J. Feng H. Sun H. Xi J. Cao Y. Li K. (2012). Functional Analysis of general odorant binding protein 2 from the meadow moth, loxostege sticticalisL. (*Lepidoptera*: *Pyralidae*). *Plos One* 7:e33589. 10.1371/journal.pone.0033589 22479417 PMC3316592

[B59] Zacharuk R. Y. (1985). “Antennae and sensilla,” in *Comprehensive insect Physiology, Biochemistry, and Pharmacology*, Vol. 6 eds Kerkut G. A. Gilbert L. I. (Oxford: Pergamon), 1–69.

[B60] Zhang S. Chen L. Z. Gu S. H. Cui J. J. Gao X. W. Zhang Y. J. (2011). Binding characterization of recombinant odorant-binding proteins from the parasitic wasp, *Microplitis mediator* (*Hymenoptera*: *Braconidae*). *J. Chem. Ecol.* 37 189–194. 10.1007/s10886-010-9902-3 21184151

[B61] Zheng Z. C. Li D. Z. Zhou A. Yi S. C. Liu H. Wang M. Q. (2016). Predicted structure of a Minus-C OBP from *Batocera horsfieldi* (Hope) suggests an intermediate structure in evolution of OBPs. *Sci. Rep.* 6 33981. 10.1038/srep33981 PMC5034290 27659921

[B62] Zhou J. J. Huang W. Zhang G. A. Pickett J. A. Field L. M. (2004). “Plus-C” odorant-binding protein genes in two *Drosophila* species and the malaria mosquito *Anopheles gambiae*. *Gene* 327 117–129. 10.1016/j.gene.2003.11.007 14960367

[B63] Zhou J. J. Robertson G. He X. Dufour S. Hooper A. M. Pickett J. A. (2009). Characterisation of *Bombyx mori* Odorant-binding proteins reveals that a general odorant-binding protein discriminates between sex pheromone components. *J. Mol. Biol.* 389 529–545. 10.1016/j.jmb.2009.04.015 19371749

[B64] Zhuang X. Wang Q. Wang B. Zhong T. Cao Y. Li K. (2014). Prediction of the key binding site of odorant-binding protein of Holotrichia oblita Faldermann (*Coleoptera*: *Scarabaeida*). *Insect Mol. Biol.* 23 381. 10.1111/imb.12088 24576058

[B65] Zwiebel L. J. Takken W. (2004). Olfactory regulation of mosquito-host interactions. *Insect Biochem. Mol. Biol.* 34 645–652. 10.1016/j.ibmb.2004.03.017 15242705 PMC3100215

