# Supplementary material for: Functional Analysis of Two Odorant-Binding Proteins, MaltOBP9 and MaltOBP10, in Monochamus alternatus Hope
Source: Front Physiol. 2020 Apr 15;11:317. doi: 10.3389/fphys.2020.00317 (PMC7174603; doi:10.3389/fphys.2020.00317)
Supplement: Supplementary file 1 [file Table_1.docx]

**Supplymental Materials**

**

**

**Figure S1.** RT-PCR amplification of MaltOBP9, MaltOBP10


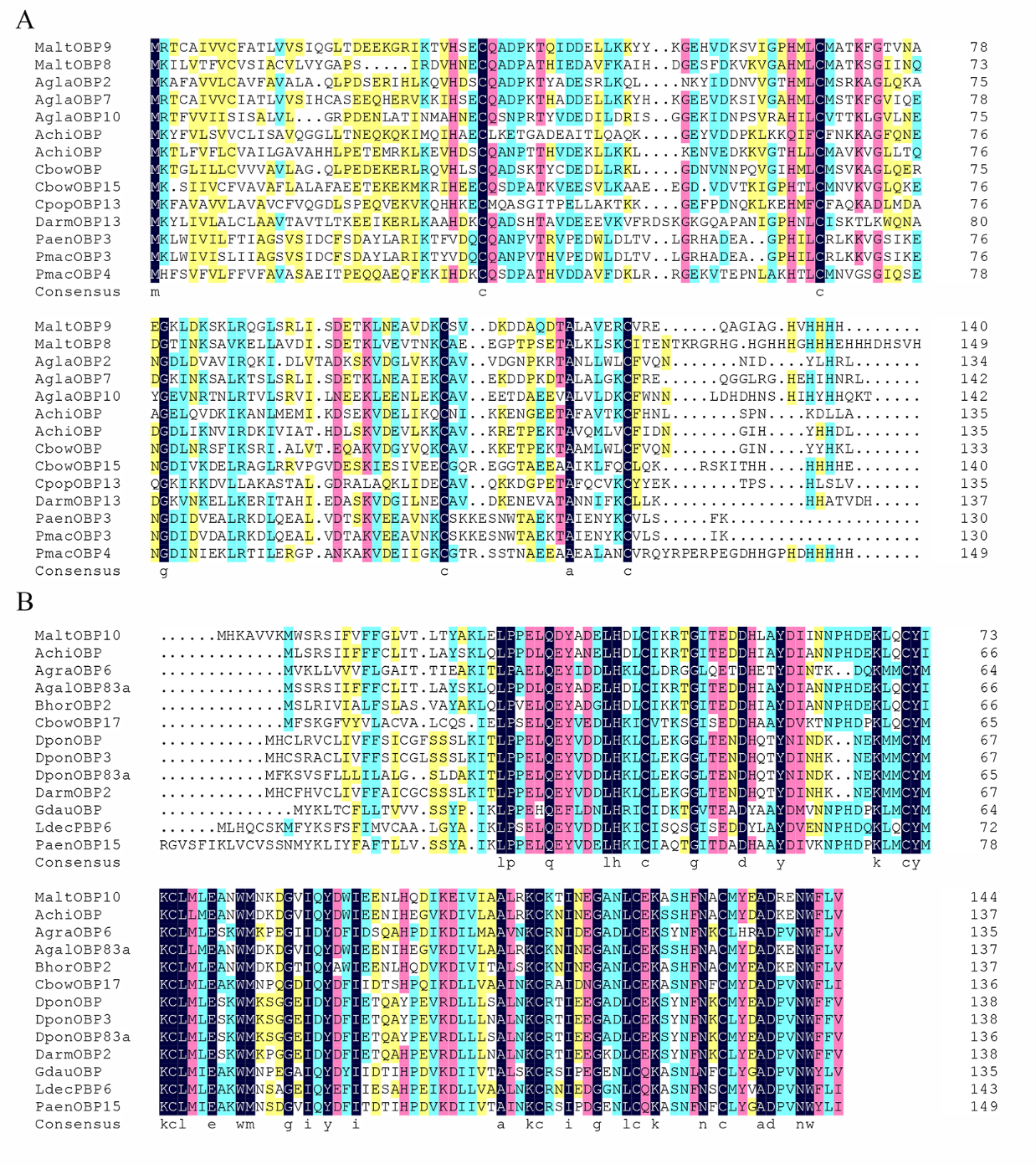


**Figure S2. Alignment of MaltOBP9 and MaltOBP10 from *M. alternatus* as well as other insects**

There OBPs shown above 33% sequence identity with MaltOBP9: *Monochamus alternatus* (MaltOBP8\ AIX97023.1), *Anoplophora glabripennis* (AglaOBP2\ ATL75740.1, AglaOBP7\ ARH65462.1, AglaOBP10\ ARH65465.1), *Anoplophora chinensis* (AchiOBP\ AUF72972.1, AchiOBP\ AUF72988.1), *Colaphellus bowringi* (CbowOBP\ ALR72494.1, CbowOBP15\ ALR72503.1), *Chrysomela populi* (CpopOBP13\ AWK23450.1), *Dendroctonus armandi* (DarmOBP13\ ALM64971.1), *Pyrrhalta aenescens* (PaenOBP3\ APC94278.1), *Pyrrhalta maculicollis* (PmacOBP3\ APC94201.1, PmacOBP4\ APC94202.1). There OBPs shown above 50% sequence identity with MaltOBP10: *Anoplophora chinensis* (AchiOBP\ AUF72991.1), *Anthonomus grandis* (AgraOBP6\ AVI04887.1), *Anoplophora glabripennis* (AglaOBP83a\ XP_023310142.1), *Batocera horsfieldi* (BhorOBP2\ AHA33380.1), *Colaphellus bowringi* (CbowOBP17\ ALR72505.1), *Dendroctonus ponderosae* (DponOBP\ AFI45061.1, DponOBP3\ AKK25131.1, DponOBP83a\ XP_019763444.1), *Dendroctonus armandi* (DarmOBP2\ AIY61045.1), *Galeruca daurica* (GdauOBP\ AQY18967.1), *Leptinotarsa decemlineata* (LdecOBP6\ XP_023024287.1), *Pyrrhalta aenescens* (PaenOBP15\ APC94288.1).
